# Application of Transformers in Cheminformatics

**DOI:** 10.1021/acs.jcim.3c02070

**Published:** 2024-05-30

**Authors:** Kha-Dinh Luong, Ambuj Singh

**Affiliations:** Department of Computer Science, University of California Santa Barbara, Santa Barbara, CA 93106, United States

**Keywords:** cheminformatics, machine learning, chemical representations, transformer, graphs, sequences

## Abstract

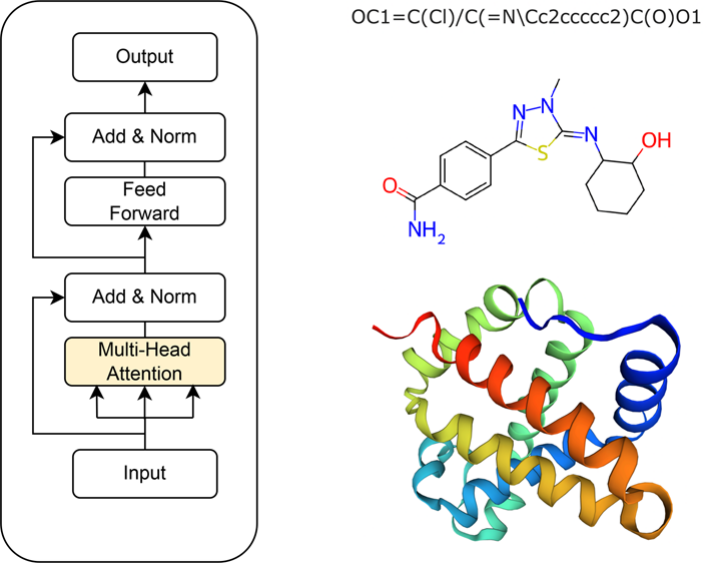

By accelerating time-consuming processes with high efficiency, computing has become an essential part of many modern chemical pipelines. Machine learning is a class of computing methods that can discover patterns within chemical data and utilize this knowledge for a wide variety of downstream tasks, such as property prediction or substance generation. The complex and diverse chemical space requires complex machine learning architectures with great learning power. Recently, learning models based on transformer architectures have revolutionized multiple domains of machine learning, including natural language processing and computer vision. Naturally, there have been ongoing endeavors in adopting these techniques to the chemical domain, resulting in a surge of publications within a short period. The diversity of chemical structures, use cases, and learning models necessitate a comprehensive summarization of existing works. In this paper, we review recent innovations in adapting transformers to solve learning problems in chemistry. Because chemical data is diverse and complex, we structure our discussion based on chemical representations. Specifically, we highlight the strengths and weaknesses of each representation, the current progress of adapting transformer architectures, and future directions.

## 1 Introduction

Chemical experiments are often expensive and time-consuming, requiring domain expertise, sophisticated equipment, and laborious operations. The long history of chemical sciences results in ample documented experimental data suitable as inputs for machine learning (ML) algorithms. By discovering useful chemical patterns within the data, ML can automate costly processes and accelerate scientific advancement. Most notably, predictive models spur the development of quantitative structure–activity relationships (QSARs) by utilizing curated labeled data sets of chemical and physical properties. Chemical generative models trained on large molecular databases help discover novel chemical structures, which is of interest in drug discovery. Utilization of ML techniques also facilitates automation in other tasks such as synthesis, experimental planning, and physical simulation.

Structural data such as chemical compounds is relatively new to ML. Earlier attempts transformed chemical structures into vectorized representations fitting traditional methods such as Support Vector Machine or Decision Tree. More recently, advanced learning architectures capable of encoding structural dependencies like convolutional neural networks (CNNs) or graph neural networks (GNNs) have been applied to learning on chemical data.^1−3^ Nevertheless, chemical structures are challenging for ML. Unlike image or text which has uniform structural patterns, chemical data contain irregular connectivities between information-rich components. As a result, there is a crucial need for complex ML methods with greater learning power to capture sophisticated chemical patterns.

Since their introduction in 2017,^4^ transformer architectures have revolutionized learning in multiple ML domains.^5,6^ Consequently, there is growing interest in developing transformer-based learning models for chemical structures. Thanks to the attention mechanism, transformers can capture long-range structural dependency, which is highly beneficial in learning complex chemical interactions. Given the diversity and structural specificity of the chemical space, this emerging field remains open to numerous challenges. Nevertheless, its potential is significant and has attracted increasing attention from the research community, leading to a substantial body of literature in a short time. To further facilitate research in this direction, we provide a comprehensive survey of existing works.

Our review of transformers in the chemical domain is structured as follows. Section 2 explains the transformer architectures and highlights related studies. After that, the review is organized around the representations of chemical data. Specifically, sections 3 and 4 delve into sequence-based representations, while section 5 explores graph-based representations. Section 6 explores the application side of transformers in cheminformatics and section 7 discusses future directions. Our objective is to elucidate the strengths and limitations inherent in different data representations as well as their unique challenges. Crucially, we highlight the innovative approaches that have been proposed to overcome these challenges. Throughout this paper, we aspire to present a valuable entry point for researchers from diverse communities, offering an insightful introduction to this intriguing area of research.

## 2 Background

### 2.1 Transformer Architecture

**Figure 1:**
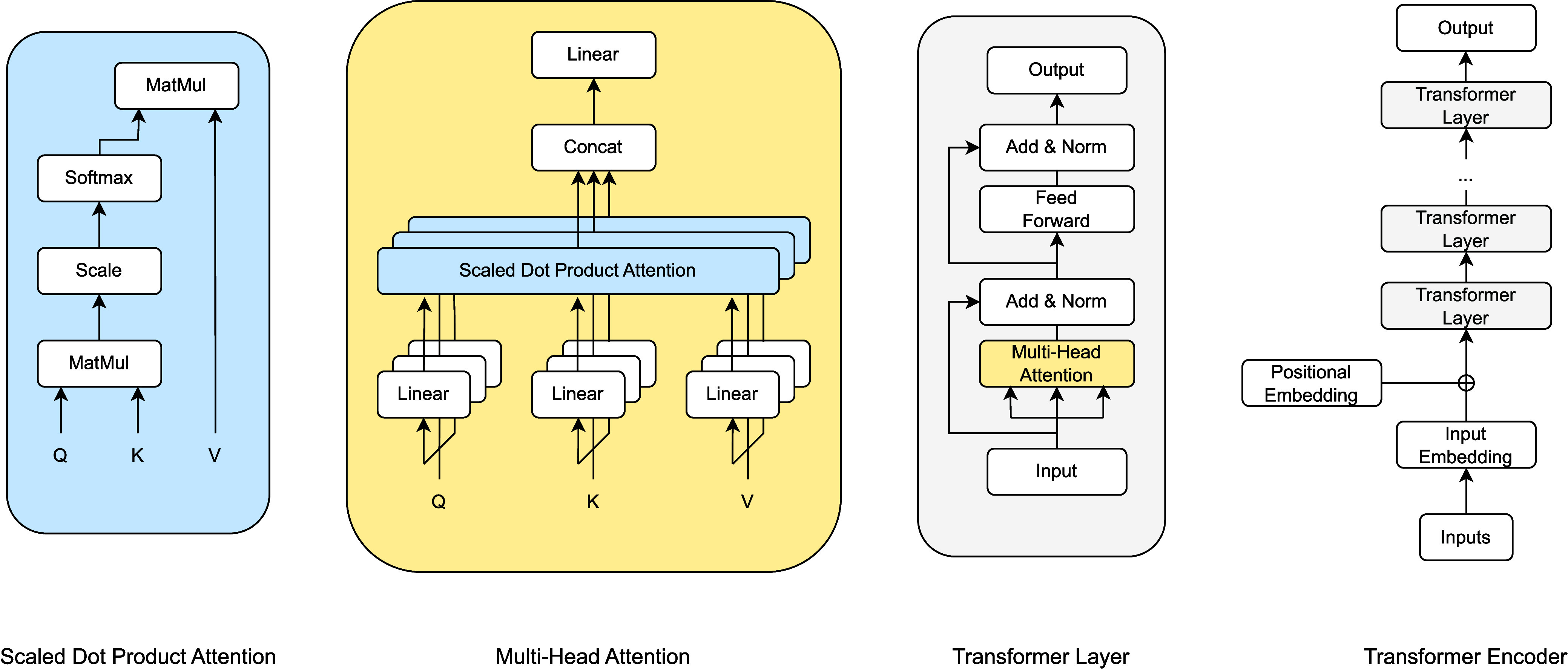
Major components of a transformer network.

Transformers are the latest advancement in deep learning, building on the success of other well-established architectures such as convolutional neural networks (CNNs) for computer vision (CV) and recurrent neural networks (RNNs) for natural language processing (NLP).^4^ The fundamental operation of transformers is simple. At each iteration or layer of the algorithm, the embedding of each element undergoes updates through referencing and combining with the embeddings of other elements. These elements can take various forms, such as tokens from a sentence, pixels from an image, or nodes from a graph. The major components and flows in a transformer network are illustrated in Figure 1.

Referencing in transformers is executed using the multihead-attention mechanism, which employs the query-keyword-value (QKV) model.^4^ This nomenclature draws inspiration from information retrieval, which focuses on assessing the relevance of a query to a given keyword. The objective of a transformer model is slightly different: we seek to determine the degree of attention, or weighting, that an element should assign when referencing another element.

Let  be the embedding of elements after the *i*th layer, where *N* is the number of elements and *D* is the input embedding size. We define three matrices , , and  as
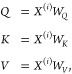
1where *D*_*k*_ is the intermediate embedding size and , , and  are separate projection heads. Let the attention matrix be , the single-head attention is calculated as
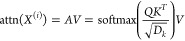
2

The attention matrix *A* contains pairwise attention weight between elements. Weights on the diagonal are regarded as self-attentions. Consequently, multihead attention concatenates multiple single-head attentions:
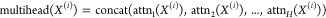
3

The embeddings of the elements are updated with residual connections that add the embeddings of the previous output:
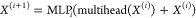
4

The attention mechanism allows each element, a word token or an atom, to reference any other elements within the same sentence or molecule. This is usually referred to as global attentions. However, if the sentence is too long or the molecule is too large, calculating the whole matrix of pairwise attentions is computationally costly. In these cases, the attention can be constrained to the surrounding neighborhood of each element, i.e, local attention. Often, additional location context is provided via positional encodings, which distinguishes elements based on their positions within the data. For example, a word token may attend differently to tokens within the same sentence than tokens from other sentences. In the original transformer,^4^ absolute positional encodings are added to the element embeddings *X*^(0)^ before feeding them into the network. Recently, relative positional encodings have been a promising approach that offers generalization to data of unseen lengths and sizes.^7^ Positional encodings are an essential part and a major challenge when adapting transformers to structural data, which we discuss more in section 5.2.

The attention mechanism, coupled with global referencing, bestows transformers with distinct advantages over other deep learning architectures like CNNs. Unlike convolutional kernels, which are static with fixed kernel weights that do not adapt to each pixel and its surrounding context, attention matrices offer dynamic learning, taking into account pairwise contextual relationships. Furthermore, the use of global attention enables an exceptionally broad perception field, surpassing the typical reach of convolutional kernels. This expanded perception field allows collecting and updating information from any element in a single step, marking a significant step up from sequential models such as recurrent neural networks (RNNs) or long short-term memory networks (LSTMs), which require as many steps as the length of the input sequence to process all elements. As a result, transformers, with the attention mechanism, are revolutionary models that consistently achieve and maintain leading positions in multiple domains of ML.

### 2.2 Transformers in Text and Image Processing

The original transformer architecture was designed for machine translation tasks. However, since the introduction of BERT,^8^ a groundbreaking transformer-based design, transformers have solidified their position as preferred architectures for a wide array of language-related learning problems. Numerous transformer-based models have since emerged, addressing challenges related to performance, generalizability, and scalability.^9,10^ Currently, modern transformer models with billions of parameters can be effectively and efficiently trained, leading to the development of large language models (LLMs).^11,12^ These pretrained LLMs possess a remarkable ability to learn from vast corpora of text using appropriate self-supervised learning strategies. Once pretrained, these models become powerful general language understanding systems, capable of performing human-like tasks, such as translation, summarization, text generation, and question answering, with human-level proficiency.^12^ When fine-tuned, an LLM easily outperforms other nonpretrained models on predictive tasks, solidifying the status of transformer-based models as state-of-the-art in NLP. Furthermore, their influence extends to various other domains of machine learning.

Transformer-based models have achieved remarkable success in computer vision (CV) and have been applied to a broad spectrum of tasks, including object detection, tracking, segmentation, and image classification.^13^ For a long time, variations of CNNs dominated the CV landscape. Transitioning from CNNs to vision-based transformers entails forfeiting some inductive bias, which is characteristic of convolutional kernels that discover generally reusable patterns, in favor of enhanced learning capabilities facilitated by pairwise context-based attentions. ViT is one of the most prominent vision-based transformers, adopting the same underlying principles as its text-based counterpart.^14^ Instead of processing word tokens, ViT divides images into gridlike patches and arranges them into an input sequence. Being a complex model, ViT is harder to train than CNNs and can outperform CNNs on large data sets but not on tasks with limited data. Recent variations of ViT, such as XCiT, PiT, LV-ViT, and DeiT, have made strides in closing this performance gap on smaller data sets by incorporating features like feature channel attentions, hierarchical feature mapping, auxiliary supervision, or knowledge distillation.^15−18^ For more comprehensive discussions on transformers in language and vision, we direct readers to existing review papers.^13^

### 2.3 Machine Learning on Chemical Structures

Compared to images and texts, chemical data exhibits greater structural complexity, posing significant challenges for ML applications. For that reason, any ML pipeline must start with identifying an appropriate representation, a decision influenced by the type of structure, the downstream task, and the chosen learning model.

For organic molecules, early efforts predominantly relied on fixed hand-crafted representations, such as descriptors and fingerprints. Descriptors are numerical or quantitative representations of various physicochemical and structural properties of the molecules. Existing descriptors can be broadly categorized into constitutional descriptors, topological and geometrical descriptors, and physicochemical descriptors. On the other hand, fingerprints are binary vectors indicating the presence or absence of predefined substructures. Fragment descriptors are similar to fingerprints as they record occurences of substructures in molecules; however, they can be nonbinary. While these vector representations are straightforward and well-suited for traditional shallow learning models, they are ineffective at capturing structural information. Consequently, there has been a growing interest in adopting string-based molecular representations like SMILES and InChI, inspired by the success of language learning models. Such string-based representations naturally lend themselves to describing biological structures like proteins and peptides, which are amino acid sequences. However, for molecular structures, the transition to string-based representations requires the incorporation of additional syntax to convert structural connectivity into text, adding complexity to the learning process. As a result, there has been a push for graph-based representation of molecules, coupled with modern graph-based learning algorithms, such as GNNs. Graphs also provide means to represent higher order protein structures and lattice patterns in material science.^187,188^

**Figure 2:**
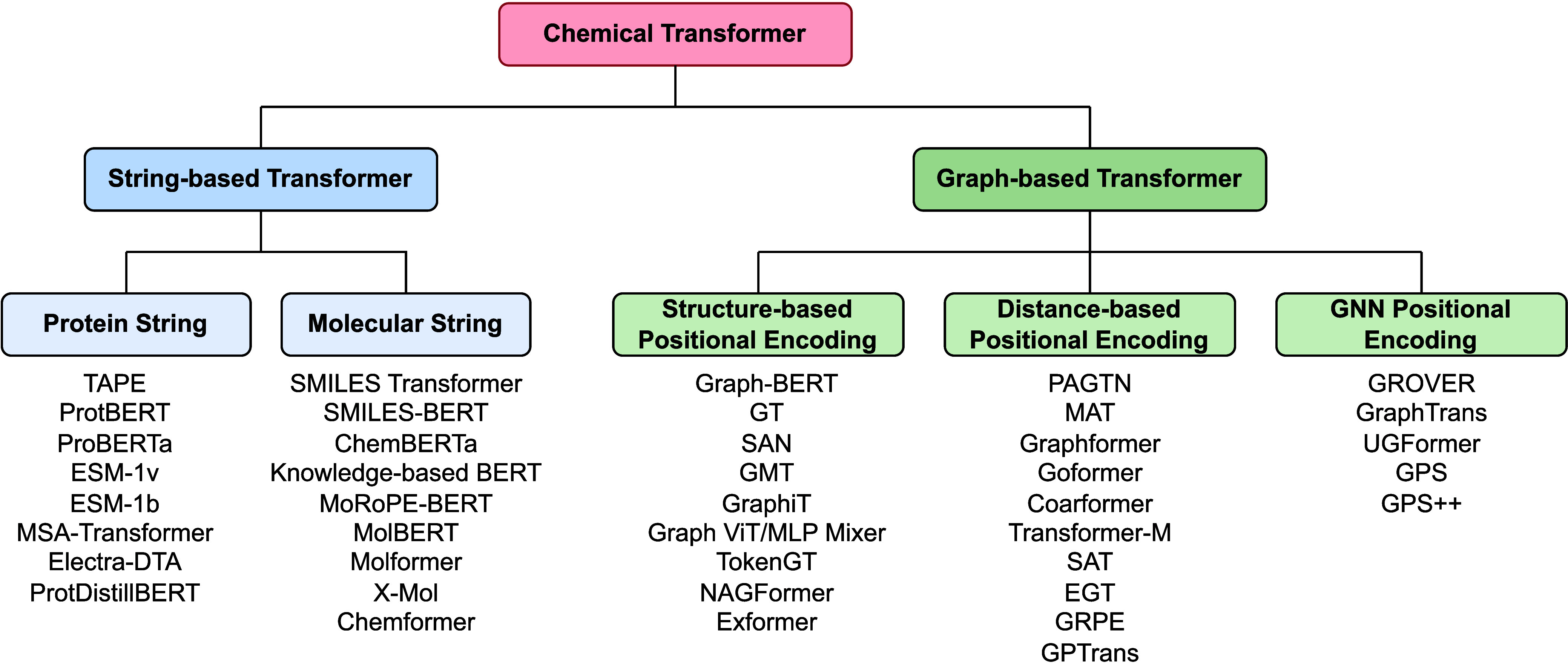
An overview of the chemical transformer landscape with a sample of methods in each category. Structure representations can be broadly categorized into either string-based or graph-based.

The rising popularity of transformers has led to a surge in research efforts aimed at adapting these models for learning with chemical structures, mostly with string-based and graph-based representations (Figure 2). The main objective of this review is to provide a comprehensive overview of these adaptations. We begin by exploring protein sequences, which resemble text data and are the most straightforward to adapt to text-based transformers.

## 3 Protein Sequences and Genomics Sequences

In this section, we review the adaptation of transformers to protein and genomics data. The representations of these data types can be easily expressed in sequential forms, making the application of transformers, developed for processing texts, more straightforward.

### 3.1 Protein Sequences

The structure of protein sequences can be likened to a complex language, where the arrangement of amino acids dictates various levels of structural folding and an array of biological functions and properties. Much like the protein space itself, these characteristics span a wide spectrum, encompassing local properties to global attributes and intrastructural states to interstructural interactions. However, determining these properties can be cumbersome. Laboratory experiments are time-consuming, and computational simulations of protein interactions may extend over hours or even days. Consequently, the prediction of protein properties and interactions from sequence information is a critical challenge that has garnered substantial interest from ML practitioners.

Given the specific challenges, transformers have proven highly adept at handling protein sequences. First and foremost, the prediction of numerous protein properties requires understanding the dependencies and interactions between components of sequences across multiple scales. The global attention mechanism, an integral feature of transformers, is tailored precisely for capturing this intricate information. Second, protein sequences closely resemble text data, an area where transformers have demonstrated remarkable success. By conceptualizing each amino acid as a word, these sequences form sentences, while higher-order protein structures can be likened to paragraphs. Lastly, the breakthroughs achieved by transformer-based models in text analysis are a result of innovative pretraining on vast text corpora. Notably, there are now open databases housing tens of millions of protein sequences, serving as invaluable data sources that allow adapting pretraining techniques that have revolutionized NLP to the realm of protein research, giving rise to protein language models (PLMs).

Exploiting the growing availability of enormous protein databases, many existing works pretrain large transformer-based language models to capture the biological syntax of amino acid sequences. TAPE and ESM-1b are among the first models to utilize transformers in learning PLMs.^19,20^ TAPE pretrains the original transformer on Pfam,^21^ a database containing thirty-one million proteins, using text-based pretraining tasks such as next-token prediction and masked-token prediction. To encode protein specificities, TAPE further supervised pretrains the model on contact prediction and remote homology prediction. ESM-1b extends training to 250 million protein sequences and conducts evaluations on a variety of prediction tasks. Given large-scale data, these papers show the superiority of transformers over earlier sequential learning models such as RNN and LSTM. In PRoBERTa, the authors optimized the RoBERTa model for proteins by pretraining using the masked-token prediction task with byte-pair encoding.^22^ Another work modifies the pretraining of RoBERTa and Longformer^23^ by injecting binding protein pairs into the training set, improving downstream performance on binding prediction tasks.^24^ ProteinBERT enriches the training of BERT by adding a novel gene ontology prediction task on top of the bidirectional language modeling.^25^ ProtTrans conducts large-scale pretraining of transformers on proteins via a combined data set of 393 billion structures.^26^ The authors experimented with a variety of transformer architectures from NLP, producing ProtBERT, ProtAlbert, ProtT5, ProtElectra, and ProtXLNet.^9,27−29^ They found that the embeddings produced by these pretrained PLMs serve as competitive initial network weights for smaller predictive models even without training. ProtDistillBERT applies a distillation technique to reduce the size of ProtBERT by half while maintaining most of its performance.^30^ Interestingly, MSA-transformer introduces a protein-specific transformer architecture that takes in a set of proteins, represented as a multiple sequence alignment, and performs novel row and column attentions on the alignment.^31^ Overall, the introduced protein foundational models based on transformers are important artifacts that can be transferred to a wide range of ML use cases.

Following the success of PLMs, transformers have emerged as a powerful means to tackle many ML problems on proteins. CollagenTransformer uses transformer-based models to predict the thermal stability of collagen triple helices.^32^ With only hundreds of downstream data points, it would be infeasible to train transformers directly from scratch. The authors exploited the pretrained ProtBERT model for the task, outperforming smaller transformer models trained directly on collagen data. Another work deploys PLMs to zero-shot prediction of the effects of mutation on the functionality of proteins.^33^ The authors introduced ESM-1v, an extension of ESM-1b, and compared it with other transformer-based PLMs such as TAPE and ProtBERT. On the basis of the BERT architecture, MutFormer is developed and pretrained on pairs of reference and mutated proteins.^34^ The model is finetuned for the prediction of missense mutation, achieving competitive performances. For drug-binding affinity prediction, ELECTRA-DTA pretrains an ELECTRA-based model to capture contextual information on protein sequences and further stacked a CNN block to capture geometrical features from the learned representation.^28^ The authors applied ELECTRA-DTA to drug repurposing and target selection for COVID-19, illustrating the capability of the model and protein transformers in general to tackle urgent emerging problems.

Arguably one of the most challenging yet crucial endeavors in chem- and bioinformatics is protein structure prediction (PSP). PSP is a long-standing problem, stemming from the interdependence between structure and functionality. The environment and the internal interactions of the 20 amino acids constituting protein sequences determine the structure of proteins. In their working environment, most proteins reliably fold into the low-energy conformation that allow them to perform their functions. Predicting the stable conformations is not only essential in understanding the characteristics of existing proteins but also in designing new proteins. As a result, performances on PSP is the standard in evaluating many PLMs.^19,20,26^ Spot-Contact-Single adapts the PLM ESM-1b to predict contact maps of protein sequences.^35^ The method notably outperforms previous models on sequences in which homologous information is limited. RGN2^36^ and trRosettaX-Single^37^ are recent PSP models on single-sequence inputs that outperform AlphaFold2^38^ and RoseTTAFold,^39^ ground-breaking graph-based PSP models that we will discuss in section 7.2, on orphan proteins and human designed proteins. In the RGN2 framework, the authors pretrained another variation of PLM called AminoBert with novel self-supervised tasks and sequence representation.^36^ trRosettaX-Single is developed upon ESM-1b and is 2 times faster than AlphaFold2.^37^

### 3.2 Genomics Sequences

Genomics sequences are another major type of biological encoding. Many discussions regarding learning on protein sequences can be applied to DNA or RNA sequences as they share significant similarities. Both can be considered languages in which sequences are constructed via constitutional units, of which the ordering determines the biological semantics and functions. However, compared to learning on protein sequences, learning transformer-based foundational models on genomics data is still an under-explored area with a potential for wide applications. As a language, genomics sequences convey rich semantic information, including those closely related to natural language such as polysemy and distant semantic relationships.^40^

The tokenization of genomics sequences is different from that of protein sequences as each token is a nucleotid base concatenated with a number of the trailing bases. Such token is called a *k*-mer if there are *k* trailing bases. Since the number of unique bases in a DNA is extremely limited, such representation helps diversify the tokens with contextual information. DNABert is the first language model on DNA sequences with the *k*-mer representations.^40^ The authors finetuned the model on multiple downstream scenarios and obtained better performances compared to those of other architectures such as CNN and LSTM. Enformer further demonstrates the superiority of transfromer architectures by training a model with a perception field of up to 100 kilobases, compared to only 20 kilobases in previous CNN models.^41^ MoDNA extends the self-supervised learning beyond *k*-mer to include repetitive motifs, improving the learned embeddings with biologically inspired patterns.^42^ Most recently, Nucleotide Transformer and DNABert-2 further introduce foundational models on genomics data.^43,44^ DNABert-2, in particular, replaces the *k*-mer representation with byte-pair tokenization, improving the efficiency and efficacy of learning embeddings.^44^

Even though transformers have been remarkable in addressing challenges related to learning from sequential protein or genomics data, it is important to note that not all types of substances can be approached similarly. While proteins or DNAs can be conveniently represented as sequences that neatly align with well-established NLP frameworks, the same does not hold for other complex structures like molecules or material lattices and adaptation in terms of the representation is often required. In the following sections, we discuss the current progress of applying transformers on inherently nonsequential structures.

## 4 Molecular Strings

Molecules constitute a major portion of the chemical space and are building blocks for larger chemical and biological structures. As a type of data, molecules are rich in information, not only from the chemical specificity of their components, such as atoms, bonds, and functional groups, but also from the connectivity and interaction between these components. As molecular structures significantly influence chemical and physical properties, preserving structural information in molecule representations is essential for extracting predictive learning patterns. Fortunately, this challenge has been a topic of interest among researchers for quite some time.^45^ However, its motivation was not rooted in machine learning but stemmed from the necessity to effectively document chemical knowledge in written text. Because of the vastness of the chemical space, it is infeasible to assign unique names to each substance. Instead, given a system of rules and syntax, each molecule can be represented as an identifying string, which in turn, can be utilized to reconstruct the molecule itself. The possibility of reconstruction means that these strings implicitly encapsulate structural information. When these representations are coupled with text-based transformer models borrowed from NLP, the prospects for advancing molecular learning become promising.

### 4.1 String-Based Molecular Representations

Numerous string-based representations have been devised for molecules, each comprising distinct sets of rules to transform molecular structures into one-dimensional strings. For instance, the IUPAC nomenclature defines a method to name organic compounds by building upon a predefined vocabulary of common substructures and functional groups.^46^ Naming becomes increasingly intricate as molecules expand in size, which requires more compact string representations, such as the international chemical identifier (InCHI) and the simplified molecular-input line-entry system (SMILES).^47,48^ Among these, SMILES is the most frequently used in text-based ML on molecules, thanks to its simple and compact expression. Since its inception in 1988, SMILES has been the standard string-based molecular representation in computational chemistry. Its ubiquity led to the development of other variations, such as BigSMILES and DeepSMILES.^49,50^

While widely adopted, SMILES is not without its limitations. Particularly, SMILES representation is not unique for each molecule. Quite commonly, multiple SMILES strings, even considerably different ones, correspond to the same molecule. Conversely, not every SMILES string translates to a valid molecule. A large portion of the SMILES space consists of such invalid SMILES.^51^ Additionally, structural differences between molecules do not translate to equivalent string edit distances in their respective SMILES representations. In other words, a minor change to a molecule may lead to a drastically different SMILES string. These phenomena pose a notable challenge to learning with SMILES as QSAR relies on using structural similarity to infer characteristic similarity. Recently, self-referencing embedded strings (SELFIES) have emerged as an innovation that mitigates some of the existing issues with SMILES.^51^ Every SELFIES corresponds to a valid molecule, ensuring the robustness of the string representation space.

### 4.2 Transformers on Molecular Strings

String-based representations of molecules recorded in existing massive accumulation of chemical documentation are valuable resources for ML, especially text-based transformer models that are extremely data-hungry. SMILES Transformer uses an encoder-decoder pipeline to pretrain a sequence-to-sequence SMILES language model.^52^ The encoder maps an input SMILES string into a latent encoding, and the decoder reconstructs the original string from this latent encoding. This encoding, which the authors called ST Fingerprint, is utilized as the input feature for shallow predictors such as support vector machines. SMILES-BERT adopts the masked language model from BERT for pretraining on SMILES, treating each SMILES string as a sentence and each atom symbol as a word token.^53^ The entire pretrained model is finetuned for downstream tasks. Compared to earlier featurizations based on molecular fingerprints, both ST fingerprint and SMILES-BERT exhibit a marked improvement in predictive quality across a range of chemical tasks. Such results motivated later works that extend pretraining to more variations of transformers, domain-specific pretext tasks, and larger-scale training data.

MolBERT pretrains BERT on SMILES with masked token prediction and auxiliary chemistry-relevant predictive tasks.^54^ These tasks include SMILES equivalent prediction, which checks whether a pair of SMILES strings encode the same molecule, and molecule descriptor prediction, which estimates various physical properties. Instead of predicting SMILES equivalence directly, knowledge-based BERT follows a contrastive setting in which embeddings of similar examples are enforced to be closer in the latent space while those of different examples are pushed apart.^55^ In this context, SMILES permutations of the same molecule form similar, or positive, pairs of examples. The authors also performed atom feature prediction from atom token embeddings and global property prediction from SMILES sequence embeddings. Interestingly, PolyBERT pretrains a language model on polymers represented as SMILES strings.^56^ TransPolymer takes a step further and defines a novel string-based representation that contains the polymer SMILES, the copolymer SMILES, and other condition parameters such as ratio and temperature.^57^

Other studies investigate the influence of large-scale pretraining on string-based molecular learning models. ChemBERTa adopts the RoBERTa pipeline for pretraining 10 million molecular strings, represented as either SMILES or SELFIES.^58^ Even though ChemBERTa was outperformed by strong existing baselines, the authors demonstrated improvement in performance with more pretraining data, strengthening the positive effect of large-scale pretraining. Recently, ChemBERTa-2, pretrained on 77 million molecular strings via a more optimized pipeline, has demonstrated competitive performance versus contemporary state-of-the-art methods on MoleculeNet benchmarks.^59^ To enforce understanding of molecular structural syntax, Chemformer pretrains BART on 100 million molecules via an autoregressive SMILES reconstruction task.^60^ Other works push the scale of the pretraining to over a billion molecular strings. X-MOL generatively pretrains various language models, BERT, RoBERTa, XLNet, T5, and ERNIE on a colossal data set of 1.1 billion molecules.^61^ Similarly, MolFormer also pretrains on 1.1 billion SMILES and introduces a masked language prediction task coupled with rotary positional encoding and a novel linear attention.^62^ These large-scale pretrained models outperform strong baselines on various downstream predictive tasks, confirming the effectiveness of transformer architectures in learning text-based chemical syntax.

Besides increasing the scale of the pretraining, other related works employ innovative mechanisms to elevate the performance of transformer models. MTL-BERT uses SMILES enumeration to tackle the data scarcity problem, especially during downstream multitask finetuning in which the authors comprehensively evaluated their models on 60 prediction tasks.^63^ MolRoPE-BERT incorporates rotary positional embeddings into pretraining BERT.^64^ Interestingly, Mol-BERT and FP-BERT utilize the masked token prediction from BERT to learn rich fingerprint embeddings. Even though they do not pretrain directly on molecular strings, these methods are inspired by Mol2Vec, which, in turn, was inspired by Word2Vec, a famous deep self-supervised pretraining method from NLP.^65−67^

## 5 Molecular Graphs

Though interesting, string-based molecular representation learning has several shortcomings. Competitive performance relies on immensely powerful transformer-based architectures, enormous unlabeled data for pretraining, and huge computing resources. The additional syntax in constructing molecular strings complicates learning, and string-based representations are not directly topologically aware. Graphs, on the other hand, can represent molecular connectivity explicitly. Incorporating chemical properties into node and edge featurizations is more straightforward in graphs compared to strings and fingerprints. As a result, graphs can be considered the most natural way to represent molecular structures. Learning on molecular graphs is an active research area in chemical ML. A large portion of state-of-the-art methods are graph-based models, such as GNNs and graph transformers.^68,69^ In this section, we review existing innovations in developing transformer-based learning models for chemical structures formulated as graphs.

### 5.1 Graph Transformers and Positional Encodings

**Figure 3:**
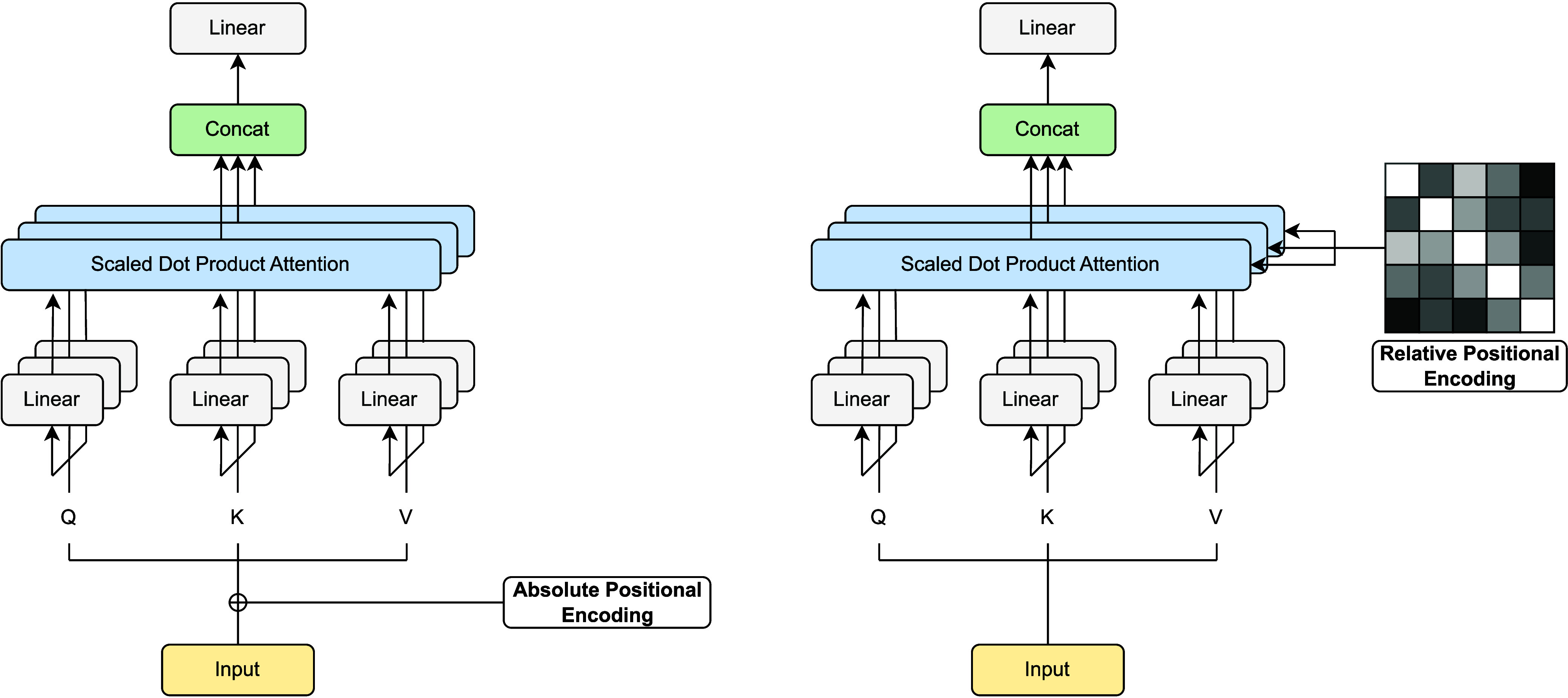
(left) Absolute positional encoding and (right) relative positional encoding. Absolute structural information can be added directly to the input node and edge embeddings. On the other hand, a common way to handle relative positional encodings is to aggregate the embedded pairwise distances to the pairwise attentions.

In an ideal scenario, it can be expected that combining the most natural molecular representations, graphs, with the most expressive learning models, transformers, would lead to a major leap in performance. However, adapting transformer architectures to graphs is a more complicated process than that of images or texts. If we consider texts as line graphs and images as grid graphs, then graphs are their generalization. Unlike line graphs and grid graphs, general graphs, including molecular graphs, do not always have uniform graph connectivity and pivoting points based on which one can define positional information. As a result, an essential problem in developing transformer-based models on graphs is effectively defining and learning graph positional encodings. In that regard, we can categorize existing techniques according to how they approach handling this problem. Specifically, we break down existing graph transformers on molecules into groups that use structure-based positional encoding, distance-based positional encoding, and positional encoding via GNNs. Despite our nomenclature, both structural-based and distance-based positional encodings capture various degrees of graph structure. While distance-based encoding focuses more on the immediate relative structural context and distance between two particular nodes, structure-based encoding encompasses the overall graph structure and the position of nodes within the context of the whole graph. Interestingly, structure-based encoding and distance-based encoding are analogous to absolute positional encoding and relative positional encoding, two common encoding paradigms for transformers on text, respectively. Figure 3 illustrates some common ways of performing absolute positional encodings and relative positional encodings.

### 5.2 Graph Transformers with Strutural Positional Encoding

Structure-based graph transformers enrich the node features and, optionally, the edge features with structural information before passing them into the network. These enrichments enhance node embeddings with graph structural information and positional information with respect to the whole graph. Essentially, nodes that are closer in the graph have similar positional encodings and vice versa. For example, Graph-BERT orders nodes via the PageRank algorithm, which obtains the ordering scores with a closed-form formula involving the adjacency matrix.^70,71^ Positional encodings are obtained from this ordering of nodes, much like in a vanilla transformer. Such encodings provide a canonical way to process a sequence of nodes; however, they fail to encode the graph structure. For this reason, the authors enhanced node embeddings with graph role embedding based on the Weisfeiler-Lehman (WL) algorithm, a standard procedure for graph isomorphism testing.^72^

Other works use the eigenvectors of the graph Laplacian matrix, which was shown to empirically perform better than WL-encoding,^73^ to capture structural and positional information.^73−77^ GT adds the Laplacian embeddings to the node features at the input layer.^73^ SAN further processes the Laplacian eigenvectors ordered by their corresponding eigenvalues via a series of linear projections and transformers before adding them to the node features.^74^ TokenGT employs a simple yet effective strategy that uses various concatenation of Laplacian embeddings to node and edge features.^76^ Graph ViT/MLP Mixer sequentially processes graph at node levels and subgraph levels.^75^ Laplacian embeddings are added to the nodes before being processed by a GNN to obtain subgraph embeddings. After that, the subgraph embeddings are enhanced with relative position embeddings obtained from the subgraph adjacency matrix weighted by the number of edges connecting pairs of subgraphs. The subgraphs are passed into a series of transformer layers and a pooling layer to obtain the final graph embedding. NAGFormer represents each node as a series of k-hop neighborhood embeddings with various radii.^77^ The Laplacian eigenvectors are concatenated to the node features before neighborhood extraction and projection. Besides the eigenvectors of the Laplacian matrix, other structure-based encoding approaches have been proposed. Graph Multiset Transformer learns to cluster nodes via the attention mechanism and uses the cluster identity as the positional encoding.^78^ Exphormer introduces attention via edges of random expander graphs generated from the input graphs.^79^

A major problem of many structure-based positional encodings, especially those relying on spectral decompositions of Laplacian matrices, is the limited transferability of these encodings between different graphs. Such a drawback is critical for inductive learning scenarios such as property prediction on molecular graphs. To tackle this problem, SAT uses random walk positional encoding, which is more transferable than Laplacian-based encoding.^80,81^ GraphiT improves transferability by exploring graph kernels defined through regularizations applied to the Laplacian spectrum and using the Gram matrix of these kernels to directly bias the attention scores.^82^ While a majority of structural encoding methods define absolute positional encoding, such a matrix acts as relative positional encoding. Biasing the attention matrix with relative positional encoding is a commonly employed strategy by distance-based methods, which we review in the next section.

### 5.3 Graph Transformers with Relative Distance Encoding

Even though relative distance encodings may not capture the overall graph structure as effectively as numerous spectral methods, their strengths reside in their simplicity and transferability. Concretely, Laplacian eigenvectors are graph-specific, and vectors from different graphs are not comparable. On the other hand, relative node-to-node distance metrics such as shortest path distance are consistent across various graphs. This property is particularly suitable for inductive learning, which may explain the recent superior performances of graph transformers with relative distance encodings on molecular prediction tasks. Additionally, the limited encoding of overall structural information may not be detrimental to these tasks since molecular graphs are quite simple. The node degrees are low due to valency, and cycles often come from relatively large aromatic rings.

PATGN encodes both the distance and the collection of edges within the shortest path between pairs of nodes.^83^ The encodings are transformed into a matrix of shortest-path features. Each path feature is concatenated to the features of the corresponding nodes to calculate the pairwise attention score. MAT biases the attention matrix with 3D interatomic distances.^84^ R-MAT extends MAT by applying the radial basis function^85^ on the 3D distance and encoding the shortest path distance between nodes.^86^ Graphormer trains a graph transformer on PCQM4M,^87^ a large predictive data set with 3.8 M molecules, and achieves a better performance over GNNs.^88^ While Graphormer also relies on shortest path encoding, unlike PATGN, the method encodes both the bonds and the bond order within each path. GRPE also encodes the shortest path; however, the model learns shortest path embeddings and uses this information to update the query and key features before calculating the attention matrix.^89^ While Graphormer uses the same relative positional bias for all layers, EGT updates the pairwise relative positional embeddings after each layer, starting with the embedding of the adjacency matrix at the input layer.^90^ GPTrans also updates the edge embeddings, employing node-to-node, edge-to-node, and node-to-edge attention.^91^ Interestingly, MolFormer defines motif nodes that connect to corresponding atom nodes that appear in the motif.^92^ The authors define attention between motif nodes and the connected atom nodes and use 3D absolute distance for relative positional encoding.

Apparently, the ever-growing interest in applying transformers to chemical graphical data has resulted in a wide variety of methods. Traversing the graph transformer landscape has become more challenging due to the sheer diversity of positional encodings, be it absolute or relative, structure-based or distance-based. GPS introduces a modular and unified framework for experimenting with various types of positional encodings.^93^ The authors also proposed a novel network layer that is a hybrid combination of the message-passing layers of GNNs and transformer layers. Such a combination is beneficial because it can leverage both the local structural encoding power of GNNs and the long-range reference capability of transformers. We review the methods that follow this line of ideas in the next subsection.

### 5.4 Combining Graph Neural Networks and Graph Transformers

Methods reviewed in this section do not necessarily require positional or structural encoding because such information can be captured by graph neural networks (GNNs). After being processed by a k-layer GNN, a node embedding would encode the structural information on its k-hop neighborhood. Note that GNNs often suffer from oversmoothing, a phenomenon associated with deeper GNNs. When a GNN has too many layers, the final node embeddings get saturated with information that embeddings of different nodes appear indistinguishable.^94,95^ Such oversmoothed embeddings may limit the quality of structural encoding. However, given enough structural diversity within a molecular graph, a moderately deep GNN can be quite useful at encoding graph positional information. When positional encoding is not explicitly defined, the network can learn to extract useful structural information without relying on hard-coded inductive bias from practitioners. Additionally, relative inductive bias may not be as useful for long-range dependencies as it is for learning short-range patterns.^96^

In place of positional encoding, several methods rely entirely on GNNs for capturing positional and structural information. In GraphTrans, a transformer module follows a GNN module.^97^ The output of the GNN module is embeddings that encode the neighborhood and structural information surrounding each node. The concatenation of these embeddings and the original node features form the inputs to the transformer module. Essentially, the embeddings produced by the GNN module replace positional encodings. Similarly, GROVER uses a dynamic message-passing network that captures neighborhoods surrounding a node with randomly sampled size before passing the output embeddings into a transformer.^98^

Instead of stacking two distinct blocks of GNN and transformer, other methods merge GNN layers and transformer layers to form hybrid layers, leveraging the advantages of both types. For example, in UGFormer, a network layer consists of a transformer layer followed by a GNN layer.^99^ Graph connectivity information is only included during the pass through the GNN layer. In contrast, GPS adopts an inverse approach in which a transformer layer follows a GNN layer. Similar to GraphTrans and GROVER, the GNN layers encode structural information. However, interweaving the GNN layers in between the transformer layers minimizes the chance of oversmoothing. The authors of GPS++ further capitalized on this idea and achieved first place on the large-scale PCQM4M benchmark for learning on molecular graphs.^100^

## 6 Applications of Transformers in Cheminformatics

Our review has focused so far on the transformer architecture and its adaptation in processing various chemical representations. For the purpose of comprehensiveness, in this section, we shift the focus to the application side of cheminformatics. We review several key areas in which machine learning has played a pivotal role with the focus on the adaptation of transformers within these domains.

### 6.1 Property Prediction

An important part of cheminformatics is the analysis of chemical activities and properties based on structural patterns, as per the development of quantitative structure–activity relationship (QSAR) and quantitative structure–property relationship (QSPR) models.^101^ These models assume a correlation between structural similarity and characteristics similarity among chemical compounds. As a result, machine learning has naturally been useful analytical tools for the development of such models. Recently, the adaptation of expressive learning architectures such as transformers has become prominent for predicting a wide array of chemical properties and activities.

The availability of benchmark databases such as MoleculeNet^102^ and Open Graph Benchmark (OGB)^87^ has played a pivotal role in advancing the development of property predictive models, especially transformer-based models spanning various modalities of molecular representations.^88,103^ Notably, OGB hosts PCQM4M and PCQM4Mv2, some of the largest labeled molecular data sets containing more than 3.8 million compounds. At the moment, graph-based transformer models such as Graphormer,^88^ EGT,^90^ and Uni-Mol,^104^ achieve the most competitive performance on these large scale challenges. In particular, Graphormer won the 2021 KDD Cup on PCQM4M and EGT currently tops the leaderboard on PCQM4Mv2.^105,106^ Most recently, long-range graph benchmark^107^ introduces several data sets on macro-molecular structures, which require the understanding of long-range dependencies among graph components. Benchmarking results on these data sets showed that transformer models outperform other learning architectures, confirming the advantage of transformers and the attention mechanism in learning complex structural interactions.

Beyond benchmark data sets, transformer-based models have been successfully applied to tackle various QSAR/QSPR problems, such as predicting solubility^108^ and toxicity.^109^ For example, Riedl et al.^110^ finetuned a pretrained language model on SMILES to predict the fraction unbound in human plasma, an important parameter in ADMET. With a similar SMILES-based model, transformer-CNN^108^ predicts AMES mutagenicity and aqueous solubility. Cremer et al.^109^ applied TorchMD-NET, an equivariant graph transformer, on predicting drug toxicity.

One important QSAR usage of learning models is the prediction of drug-target affinity prediction (DTA). DTA is a crucial step in computer-aided drug discovery since it helps identifying appropriate drug targets, usually physiological proteins, and the design of molecules capable of modulating their activity. Transformers are well-suited for this task as their attention mechanism not only allow effective capturing of drug-target interactions and long-range structural information, but also result in more interpretability. For that reason, there has been increasing adaptations of transformers for DTA. For example, DTITR^111^ employs transformer encoders to process SMILES strings and protein sequences, followed by a cross-attention module to produce molecule-protein interaction embeddings. Another work by Kang et al.^112^ adapts additional BERT-like pretrainings on the transformer encoders separately for either SMILES strings or protein sequences. TEFDTA^113^ extends the analysis to bonded (valence) interactions. The authors represented molecules as MACCS fingerprints. A transformer encoder processes these MACCS vectors while a 1D convolutional neural network processes the protein sequences. TAG-DTA^114^ is another transformer-based model on SMILES strings and protein sequences in which an auxiliary binding pocket prediction task is learned in parallel in order to condition and guide the main DTA task. Other works look into interpretability. MolTrans^115^ is a popular model that works on SMILES and protein subsequences. The authors computationally mined common SMILES and protein subsequences from large databases and decomposed input SMILES or proteins based on the mined vocabulary of subsequences. These sequences of substructures are processed by transformer encoders and pairwise interaction scores between each pair of protein substructure, and a molecular substructure is obtained via dot products of the embeddings. The resulting interaction map casts light on the engagement between drugs and targets. HoTS^116^ pretrains the encoders on complexes of protein–ligand interaction. Evaluated interpretability of downstream DTA predictions are obtained from the attention scores. To better capture structural information, which is essential for DTA, several recent methods such as GTAMP-DTA,^117^ AttentionMGT-DTA,^118^ and TransVAE-DTA^119^ turn to graph representations, representing molecules and proteins as molecular graph and protein pocket graphs and processing them via graph transformers. These models obtain strong performances on DTA predictions.

### 6.2 Structure Generation

Generative models are at the forefront of AI research, encompassing diverse domains including chemical structures. Out of the 10^23^ to 10^60^ possible druglike molecules,^120,121^ only a minuscule portion has been synthesized,^122^ explaining the strong need for the discovery and generation of new drug candidates. As a result, generative models have been developed in parallel with predictive models as a major part of machine learning in cheminformatics. Coupling generative and predictive methods allows the discovery of chemically valid molecules with one or multiple desired properties. Existing generative models span a wide variety of paradigms, including variation encoders, generative adversarial networks, flow-based models, transformer-based models, and diffusion.^123^ Given the prevalence of transformers in the generative AI landscape, it is compelling to explore their potential in discovering novel chemical structures.

Text-based representations such as SMILES are common choices for chemical generative models because of the natural transferability of successful transformer-based language models. For instance, GMTransformer^124^ autoregressively generates molecular strings via pretraining a transformer network with a novel blank filling language model. The authors experimented with various string-based representations, including SMILES, SELFIES, and DeepSMILES. Regarding generation of molecules with target properties, MCMG^125^ trains a conditional transformer with reinforcement learning and knowledge distillation. The model effectively generates molecules with multiple desired properties. In a different flavor, CMGN^126^ autoregressively reconstruct molecules from fragments, conditioning on certain properties. RegressionTransformer^127^ is another conditional transformer model that not only generates molecules with high-quality continuous attributes but also outperforms multiple baselines on regression tasks. Besides string-based representations, MolFormer^92^ explores the generation on molecular graphs.

Several methods utilize the ability of transformer in interdomain translation to train generative models. Grechishnikova^128^ trained a transformer model to translate from amino acid sequences to SMILES, effectively generating molecules for given target proteins. TransAntivirus^129^ translates IUPAC nomenclatures into SMILES. This translation includes select-and-replace edit that transform input molecules into ones with desired properties, with the application in discovering antiviral compounds. In general, translation is a powerful mechanism of transformer architectures with, beyond structure generation, many important use cases in cheminformatics, which we review in the following section.

### 6.3 Chemical Translations

The original transformer was developed for the machine translation task,^4^ which converts texts from one language to those with the same meaning in another language. However, this mechanism can be applied to use cases outside of the ordinary language translation. An example is in the question answering task in which the input question is “translated” into the appropriate answer.^130^ In general, translation via transformer can be extended to mapping between multimodal data or data distributions. This usage is especially applicable for chemical data in which multiple representations often exist for the same compound. For instance, the transformer model on natural language text can be readily adapted to translating from SMILES to IUPAC^131^ or from InCHI to IUPAC.^132^ Notably, pretraining a transformer model on the SMILES-to-IUPAC translation task has been found to result in better performance when finetuned on downstream tasks, such as binding affinity prediction.^133^ Another interesting application is in optical chemical structure recognition, in which the chemical structure information on compounds from scientific records and publications is converted into machine readable formats. Several works formulate this task as an image-to-text translation with the text being any string-based representation of chemical structures.^134−136^ For instance, SwinOCSR^134^ uses a Swin Transformer for image to SMILES translation. Other similar models include Image2Smiles^135^ and DECIMER.^136^ Interestingly, MassGenie^137^ predicts chemical structures from mass spectroscopy by translating the mass spectroscopy of molecular fragments into the original molecule.

Analogical to the question answering task, in cheminformatics, translation can be applied to generate “answers” to a given “question”, both represented as chemical structures. For example, to generate druglike molecules that target specific proteins, several works translate input amino acid sequences into SMILES strings of molecule with appropriate binding activities.^125,128^ To optimize existing molecules in order to obtain certain desired properties, translation models are trained to make adjustment to input molecules or scaffold. He et al.^138^ trained a conditional transformer model that converts an aggregated input of the source molecule SMILES string and the target property into an output SMILES string of a molecule possessing the property. Similarity constraints ensure that the output molecule closely resemble the source molecule. DeepHop^139^ uses translation to perform scaffold hopping in which the output molecule is novel in terms of scaffold as compared to the input molecule while maintaining similar bioactivity, which is ensured via enforcing 3D structural similarity. MetaTrans^140^ translate an input molecule into its products after going through various metabolism procedures, effectively predicting metabolism outcomes of drugs.

### 6.4 Chemical Reactions

Chemical reaction analysis is another pivotal domain within cheminformatics where computational methodologies and learning techniques have made significant strides. Both the forward problem, entailing the prediction of reaction outcomes, and the backward problem, involving retrosynthetic analysis, have witnessed substantial advancements aided by modern learning architectures. While earlier approaches rely upon rule-based procedures and reaction templates, of which the extraction from existing literature is a laborous and tricky process, recent learning-based methods explore template-free modeling of chemical reactions. In these template-free models, chemical reaction information is automatically learned from data and stored as embeddings in deep neural networks, resulting in more flexibility and robustness of both the forward and backward analysis. The rise of transformers has catalyzed modeling chemical reactions as a translation problem. Intuitively, this formulation fits the idea of chemical reaction being a transition of a chemical system from the reactants to the products and vice versa.

A prime example of formulating reactions as translations is the Molecule Transformer,^141^ a popular translational model for predicting reaction outcomes. The authors modeled the mapping from the reactants to the products as a SMILES-to-SMILES translation. Molecule Transformer outperforms previous baselines by large margins on various experimental settings. Jaume-Santero et al.^142^ further investigated the effects of different training parameters, including representations (SMILES or SELFIES), tokenization (atom or byte-pair encoding), pretraining, data augmentations, and predictive tasks, on the performance of Molecule Transformer. Pesciullesi et al.^143^ developed finetuning strategies for Molecule Transformer and evaluated the model on predicting regio-stereoselective reactions on carbohydrates. Andronov et al.^144^ used the translation formulation to predict reaction reagents. In this case, the input is the whole reaction string instead of just the reactants. A few recent works incorporate both the graph and string representations into the translation process.^145,146^ Graph2SMILES^145^ translates the molecular graphs of reactants into the SMILES strings of products. The authors replaced the transformer encoder with a graph neural network (GNN). Instead of GNN, SeqAGraph^146^ develops a novel transformer-based graph encoder that assigns node ordering on the graph atoms based on the corresponding SMILES string. The whole model is trained for both forward and backward (retrosynthesis) prediction problems.

Similarly, translational transformers are also popular for the backward retrosynthetic analysis of chemical compounds. For instance, Karpov et al.^147^ trained a translational transformer model that converts the SMILES string of a compound into another SMILES string of its constitutional reactants. Schwaller et al.^148^ coupled the single-step backward prediction with hyper graph representations of chemical reactions to discover synthetic pathways. SCROP^149^ couples the translational transformer with a neural network-based syntax corrector, significantly reducing the number of chemically invalid candidate precursors. Tetko et al.^150^ employed data augmentation on the input SMILES strings, noticeably improving the performance of their model across multiple metrics. RetroPrime^151^ disects a retrosynthesis step into 2 steps: generating sythons and adding leaving groups. The authors developed 2 separate translational models for these steps. Recent methods on retrosynthesis also incorporate molecular graph information on top of the SMILES representation. For example, RetroFormer^152^ has separate attention heads for the global attentions on SMILES strings and the local attentions that encode neighborhood connectivities on molecular graphs. The aggregated embeddings of these attentions serves as the input to the decoder, which outputs SMILES strings. G2GT^153^ takes a step further and formulate retrosynthesis as a graph-to-graph translation problem in which they utilize the Graphormer architecture^88^ as the encoder and the decoder. The method outperforms other template-free transformer baselines on top-1 accuracy.

Transformer-based models also have other interesting applications within the chemical reaction domain. For example, Wang et al.^154^ introduced the reaction generation task and trained the Transformer-XL to generate novel reactions within the same reaction class. Thousands of generated reactions were assessed and confirmed by chemists, and the whole process from training to confirmation took only 15 days. GraphormerMapper^155^ trains a atom-mapping model based on the BERT architecture with the encoder replaced by a Graphormer encoder.^88^ The model effectively maps corresponding atoms between the reactants and the resulting molecule from chemical reactions, outperforming the state-of-the-art atom mapping algorithm. In a different flavor, Schwaller et al.^156^ used the attention weights of a pretrained transformer model to capture the atom mapping. Another work by Schwaller et al.^157^ predicts and analyzes reaction classes. They trained a SMILES-to-SMILES translational transformer model in an unsupervised manner and a BERT-based encoder-only model in an supervised manner on reaction classification. Both models achieve remarkable classification performance. Additionally, the authors found that the embeddings learned by these models, which they termed reaction fingerprints, effectively capture the clustering of various chemical reaction classes with granular details and differences.

## 7 Future Directions

We conclude the review on the application of transformer-based architectures in cheminformatics by discussing interesting research directions. These directions encompass novel applications and developments of models capable of encoding domain-specific, geometric, and multimodal information. Such approaches potentially achieve improved performances on existing tasks and solving more complex ones.

### 7.1 Transformers for Molecular Dynamics Simulations

We have witnessed rapid progress on the analysis of proteins, especially on crucial tasks such as protein structure and protein-drug affinity predictions. However, as encouraging as they are, these tasks present only interactions under fixed conformations. Existing learning frameworks provide limited capability in reasoning about dynamic chemical/biological configurations. One important use case of such reasoning is in targeting protein misfolding and degenerative diseases.^158^ Degenerative diseases such as Alzheimer’s and Parkinson’s are extremely common, affecting millions of people worldwide, and are directly linked to the misfolding of certain proteins.^159,160^ Developing prevention and treatment for these diseases requires understanding the effects of drugs on flexible protein conformations.

Molecular dynamics (MD) simulations can cast light on interactions and changes in protein structures; however, they are costly in terms of time and computation and not suitable for large scale screening of drugs. Machine learning has the potential to automate and speed up parts or the whole process. Moreover, transformer-based models are particularly suitable for simulations since they can effectively capture multiscale interactions between drugs and protein sequences amd handle multimodal data. For example, Wang et al.^161^ formulated molecular dynamics simulations as a generative problem with the goal of discovering novel conformation. A transformer encoder-decoder network is trained to predict propagating frames of protein complexes with data obtained from MD simulations. Despite its importance, this direction is still quite unexplored with only a few other works with similar ideas.^162−164^ A general approach toward generative models using MD simulation is still much desired.

### 7.2 Equivariant Graph Transformers

Despite their usual 2D visualization, molecules do not lie on the 2D space as planar graphs. Instead, a large portion of their chemical properties depend on their 3D geometrical alignments and interactions. As a result, more works have investigated the incorporation of 3D geometric features into the learning of molecular graphs. However, such a process is not straightforward. Due to translations and rotations, there can be an intractable number of equivalent 3D atomic coordinates to the same molecule. Translation and rotation form the SE(3) group in the 3D Euclidean space, and as such, geometric learning on molecules requires a certain degree of equivariant or invariant with respect to SE(3) transformations.

Before transformers, several GNNs have experimented with equivariant convolution. Inspired by the use of Clebsch-Gordan coefficients and the spherical harmonics in Tensor Field Networks,^165^ SE(3)-Transformer connects SE(3) equivariant and the attention mechanism.^166^ In particular, the method achieves roto-translational equivariance within the node embeddings and invariance with respect to the attention weights. TorchMD-Net obtains rotational equivariance by encoding interatomic distances via the radial basis functions.^167^ GPS++ encodes interatomic distances with Gaussian kernels.^100^ Besides rotation and translation, Equiformer extends equivariance to inversion. The authors replaced the dot product attention with multilayer perceptron attention and nonlinear messages, leading to higher expressive power.^168^

By extending geometric learning to graph transformers, equivariant transformers allow fuller utilization of the long-range referencing via the attention mechanism. Such property is especially beneficial to tasks in which 3D geometric information is important, such as energy, binding affinity, and protein folding prediction. For example, RoseTTAFold, a recent remarkable work on protein folding prediction uses SE(3)-Transformer as the backbone of the pipeline.^39^ Similarly, AlphaFold2, a revolutionizing model for protein folding prediction, is backed by an original equivariant graph transformer layer called Evoformer and novel triangle attentions.^38^ AlphaFold2 was the top performer on the CASP14 protein folding challenge and is the state-of-the-art model. These facts signify the importance of equivariant transformers as a research direction in geometric learning and ML within the chemical domain.

### 7.3 Graph Transformers for Infinitely Repetitive Patterns

Graph-based learning on material structures is an interesting direction that has recently captured increasing attention. These graphs consist of local connectivity patterns between atoms within a unit cell and global connectivity patterns among an infinitely repetitive lattice of unit cells. These properties pose a significant challenge in constructing graphical representations for such data. With the availability of larger material data sets such as the Material Project or the Cambridge Structural Database, there is an interest in applying complex learning models to solve learning problems in this data domain.^169,170^

CGCNN is an earlier work that tackles this problem.^171^ For any crystal lattice, the authors constructed a compact graph in which nodes are atoms in the unit cell and edges represent both intercell and intracell connectivities. Since lattice data does not often contain explicit bonding information, connectivities are determined via a distance threshold. Matformer further capitalizes on this graph construction and rigorously proves its periodic invariance.^172^ They enriched the attention mechanism with edge distances featurized via radial basis functions, achieving roto-translational and reflective invariance. MOFNet works on metal–organic frameworks (MOF) and only applies transformer-based embedding on the local graph representation of the unit cell.^173^ For capturing global lattice patterns, MOFNet incorporates features such as crystal density, porous volume, gravimetric surface area, etc. Xtal2DoS learns to predict the density of states of crystals.^174^ The authors used GAT, an attention-based GNN,^175^ to embed both local and global patterns, then employed a transformer-based model to decode the embeddings to density sequences. To further advance performance, other methods attempt pretraining MOF on large data sets. In a supervised manner, MOFTransformer pretrains a standard transformer model to predict the topology, the void fraction, the metal cluster, and the organic linker of more than 1 million hypothetical MOF.^176^ The pretrained model captures both local and global features of MOF lattices and obtains competitive results on downstream tasks such as gas absorption prediction. Instead of supervised pretraining, MOFormer pretrains in a self-supervised manner.^177^ The authors used GCGNN to learn node embeddings that capture the graph connectivity of unit cells and a text-based transformer that processes the string-based representations of MOFs. The framework then contrastively enforces the correlation between both embeddings.

### 7.4 Multimodal Pretraining with Large Chemical Knowledge Bases

Despite the availability of large chemical knowledge bases,^122^ most existing methods only extract patterns from molecular structures, neglecting an abundance of corresponding chemical information stored as text descriptions. The main challenge of exploiting these chemical corpora is the multimodality of the data, i.e, graphs and SMILES strings versus natural language texts. Many recent works take on the challenge by leveraging the powerful processing ability of transformer-based language models.

Joint learning of SMILES strings and texts is an apparent direction because both modalities can share the same learning architecture and utilize NLP learning techniques. For example, KV-PLM^178^ learn to process both SMILES strings and text descriptions using the masked token prediction task and the BERT model.^8^ The model processes molecule text descriptions with masked tokens being either byte-pair encoded SMILES strings placed next to the substance name in the text or other randomly selected words. The pretrained model then performs tasks such as property or reaction prediction using SMILES strings as inputs. Similarly, MolT5^179^ trains a single medium-size T5 model for both text descriptions and SMILES strings using the replace-disrupted-span objective. Downstream tasks include molecule captioning which translates SMILES into text and text-to-molecule generation which outputs SMILES according to textual descriptions. MolXPT^180^ replaces substance names with SMILES strings and pretrains on the combined corpus of texts, texts with wrapped SMILES, and SMILES strings. The downstream predictions on molecular properties are obtained via prompting. MolReGPT^181^ employs LLM to perform molecule and text translation.

Other works expand the multimodal learning to other molecular representations. Text2Mol^182^ uses molecular graphs with Mol2Vec^66^ embeddings as node features. The embeddings of these graphs produced by a GNN are contrastively pretrained against the text embeddings produced by SciBERT,^183^ a language model trained on scientific texts. Following a similar approach contrasting texts against molecular graphs, MoMu^184^ extensively evaluates the pretrained model on a variety of challenging downstream tasks, such as cross-modal retrieval, molecule captioning, zero-shot text-to-molecule generation, and property prediction. Interestingly, CLAMP^185^ finds that traditional fingerprints work better than SMILES or graphs in representing molecules for contrastively pretraining against texts. Finally, GIT-Mol^186^ pretrains on a wide range of modality including texts, images, and graphs.

### 7.5 Large Language Models

Recently, the most important artifacts built upon the transformer architecture are undoubtedly large language models (LLMs). LLMs such as GPT, Llama, Claude, and Falcon have significantly impacted various fields, including technology, education, creativity, business, healthcare and the society at large.^189^ The revolutional power of LLMs comes from their ability to efficiently process enormous text corpus, capture the underlying associations/patterns, and reproduce the information via an intuitive interface with human-like communication. Driven by this wave of success, there is an rising interest in applying LLMs to assist scientific research. Most recent findings on the usage of LLMs in cheminformatics show that this is a promising direction.

The most straightforward application is using LLMs as property predictors via smart prompting.^190^ In ChemLLMBench,^191^ the authors created a testbed for benchmarking LLMs with eight chemical property prediction tasks. Jablonka et al.^192^ finetune GPT-3 model on molecular classification and regression tasks. More importantly, the authors attempt inverse designing by training the LLM to generate molecular photoswitches with a desired range of wavelength. Although most generated molecules belong to the training set, a considerable portion of them are novel and do not exist in PubChem. Further evaluation reveals that the mean absolute error on the transition wavelengths of these molecules is remarkably around 10 percents of the desired values. ChemCrow^193^ integrates existing chemical tools with LLMs to improve chemical research. In particular, chemical tools can help with input processing and output correction. Using such hybrid approach enhances the overall performance of various chemical tasks, including property prediction, structure generation, and prediction outcome forecasting. Interestingly, White et al.^194^ formulate chemistry problems as coding tasks, on which LLMs have been shown to perform well. More specifically, the inputs are incomplete code with instructions as comments and LLMs are asked to complete the code to produce a function that executes certain chemical calculations.

As a new direction, applying LLMs on chemical tasks still requires further research and development. Encouragingly, the scientific community has responded with enthusiasm. In a relatively short period, numerous benchmarks and competitions have been established to expedite progress in this area.^191,195^ We anticipate further advancements that will significantly enhance computational methods in cheminformatics.

## 8 Summary and Conclusion

Machine learning models play an important part in many modern chemical pipelines as they can efficiently assist or even replace expensive and time-consuming chemical experiments. This trend is assisted by the growing availability of large chemical databases and the rapid development of machine learning methods. Even though learning algorithms are traditionally developed for tabular and vectorized data, there has been a growing number of methods geared toward structural data, such as graph neural networks. These methods have led to a surge of applications and advancements in terms of performance on multiple chemical learning tasks. This fact confirms the effectiveness of powerful structural learning architectures on complex data in the chemical domain. For that reason, transformer models, which recently revolutionized learning in natural language processing and computer vision, gained the attention of researchers as a potential solution to chemical learning problems. In this paper, we reviewed recent efforts in applying transformer architectures to learning in the chemical domain.

Many methods utilize the 2D sequential representations of chemical structures to fit the input configuration of transformers. Examples include string-based amino acid chains, SMILES, and SELFIES. Such setup conveniently benefits from the established transformer models developed for text processing. Since text data are simple to obtain and process, large-scale self-supervised learning is feasible, resulting in multiple foundational models for string-based chemical data. Other works adapt and develop novel transformer architectures fitting the intrinsic representation and properties of chemical data, often in the form of graphs. A wide variety of graph transformers have been developed for this purpose, taking into account multiple geometric and chemical characteristics of structures. Interestingly, several research directions focus on domain-specific characteristics, such as equivariant transformers that are invariant to equivalent 3D configurations or graph transformers that process infinitely repetitive patterns to learn on material lattices.

Overall, transformer models are highly capable learning architectures, and the existing methods we review show the potential of transformers on chemical data. The chemical domain is vast and diverse with a variety of structures, a wide range of problems, and a diversity of physical and chemical characteristics that can be exploited for learning. As research efforts continue to rapidly expand across multiple scientific communities, we have great confidence that even more exciting results await us in the near future.
